# Paper-Supported High-Throughput 3D Culturing, Trapping, and Monitoring of Caenorhabditis Elegans

**DOI:** 10.3390/mi11010099

**Published:** 2020-01-17

**Authors:** Mehdi Tahernia, Maedeh Mohammadifar, Seokheun Choi

**Affiliations:** Bioelectronics & Microsystems Laboratory, Department of Electrical & Computer Engineering, State University of New York-Binghamton, Binghamton, NY 13902, USA; mtahern1@binghamton.edu (M.T.); mmoham11@binghamton.edu (M.M.)

**Keywords:** *C. elegans*, paper-based platforms, transparent polycarbonate substrate, 3D culturing environments, high-throughput

## Abstract

We developed an innovative paper-based platform for high-throughput culturing, trapping, and monitoring of *C. elegans*. A 96-well array was readily fabricated by placing a nutrient-replenished paper substrate on a micromachined 96-well plastic frame, providing high-throughput 3D culturing environments and in situ analysis of the worms. The paper allows *C. elegans* to pass through the porous and aquatic paper matrix until the worms grow and reach the next developmental stages with the increased body size comparable to the paper pores. When the diameter of *C. elegans* becomes larger than the pore size of the paper substrate, the worms are trapped and immobilized for further high-throughput imaging and analysis. This work will offer a simple yet powerful technique for high-throughput sorting and monitoring of *C. elegans* at a different larval stage by controlling and choosing different pore sizes of paper. Furthermore, we developed another type of 3D culturing system by using paper-like transparent polycarbonate substrates for higher resolution imaging. The device used the multi-laminate structure of the polycarbonate layers as a scaffold to mimic the worm’s 3D natural habitats. Since the substrate is thin, mechanically strong, and largely porous, the layered structure allowed *C. elegans* to move and behave freely in 3D and promoted the efficient growth of both *C. elegans* and their primary food, *E. coli*. The transparency of the structure facilitated visualization of the worms under a microscope. Development, fertility, and dynamic behavior of *C. elegans* in the 3D culture platform outperformed those of the standard 2D cultivation technique.

## 1. Introduction

Growing small organisms such as human cells and bacteria under carefully controlled conditions, generally outside their natural habitats, is a key step for tissue engineering, drug screening and development, and disease modeling [1]. Cultures have been mainly carried out on 2D platforms, such as petri dishes, microtiter plates, and flasks, in which the organisms are placed onto a flat surface that is different from in vivo 3D environment [2]. Although the conventional 2D culture techniques generated a wealth of new scientific and technological results with significant and transformative potential, they fail to provide an accurate structure, function, or physiology of living organisms and even worse can alter their gene expression, metabolism, production, and morphology compared to those in 3D [1,2,3,4]. These organisms inherently respond to a variety of chemical and physical cues from their 3D microenvironment differently than 2D cues while performing the fundamental cellular processes such as proliferation, differentiation, apoptosis, and migration. Therefore, mimicking the 3D environment is required to stimulate the actual in vivo growth conditions, provide an accurate model of the living organisms, and thus revolutionize bioengineering and biomedical applications. 

Among many small organisms, *Caenorhabditis elegans* is widely used as a model to study mechanisms of neurobiological processes and human diseases [5,6]. As many of the cellular and molecular processes in *C. elegans* have proven relevant for human biology, the worm has been an important model system to study the mechanisms of disease and perform drug discovery experiments. However, *C. elegans* have been notoriously unable to be manipulated in the 3D microenvironment. There are technical challenges to control nutrient and gas transport in 3D and provide a 3D scaffold for *C. elegans* having different sizes in terms of their developmental stages (egg, L1, L2, L3, L4, and adult stages). Developmental transitions in *C. elegans* and their reproduction efficiencies rely extensively on culturing conditions. Typically, *C. elegans* is cultured using the *E. coli* strain OP50 as a food source on a standard 2D Nematode growth medium (NGM) agar [7]. However, the worms cultured on a 2D plate are not accurate models of those in their in vivo environments such as rotting fruits and vegetation, and there are limitations in understanding their 3D locomotion behavior at different life stages during cultivation. Therefore, there is a need to develop 3D culture methods for *C. elegans*. High-throughput 3D cultivation of *C. elegans* can be a more powerful technique to identify various genes or drugs potentially relevant to human biology or diseases [8]. Additionally, their mechanical immobilization in their physiologically active state is beneficial for high-resolution microscopic monitoring [9,10]. However, reported work on the 3D culture technique with trapping and monitoring of *C. elegans* in a high-throughput manner is quite limited. High-throughput culturing tools and trapping methods for *C. elegans* have historically developed separately, and the techniques developed for those applications are usually incompatible with each other [11,12]. Even the latest advances in a 3D controlled environment do not accurately replicate their natural habitats and do not show any significant difference in culturing compared to the standard 2D method [3]. 

Here, we created a new 3D, high-throughput technique to accomplish all culturing, trapping, and monitoring functions for *C. elegans* by using paper as a 3D scaffold. A 96-well array was readily fabricated by placing a nutrient-replenished paper substrate on a micromachined 96-well plastic frame, providing high-throughput 3D culturing environments and in situ analysis of the worms. Paper allows *C. elegans* to pass through a porous and aquatic matrix until the worms grow and reach the next developmental stages with the increased body size comparable to the paper pores. When the diameter of *C. elegans* becomes larger than the pore size of the paper substrate, the worms are trapped and immobilized for further high-throughput imaging and analysis. To facilitate visualization of the works, we also constructed another type of culture system by using paper-like transparent polycarbonate substrates. The system used a multi-laminate structure of thin polycarbonate layers as a scaffold to mimic the worm’s 3D natural habitat. We observed that development, fertility, and dynamic behavior of *C. elegans* in the 3D culture system outperformed those of the standard 2D cultivation technique. 

## 2. Results and Discussion

The integrated technology for cultivation, immobilization, and monitoring of *C. elegans* will help promote barrier-transcending and translational advances in studying them. Recently, paper has been used as a substrate for 3D cell culturing and high-throughput biological and chemical analysis because of its intrinsic advantages including low cost, disposability, and easy fabrication [13,14,15]. Moreover, paper is attractive because it wicks fluids without external pumps and tubes [15]. In addition, paper’s well-defined pore sizes can filter to separate different-sized molecules from samples [16,17]. Despite its vast potential, the promise of paper has never been used for *C. elegans* studies. 

### 2.1. High-Throughput Culturing, Trapping, and Monitoring of C. elegans

**Figure 1:**
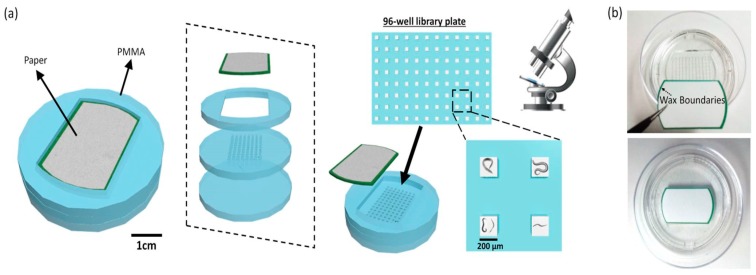
(**a**) Schematic diagrams and (**b**) photos of the proposed paper-based culture platform *for C. elegans* studies.

**Figure 2:**
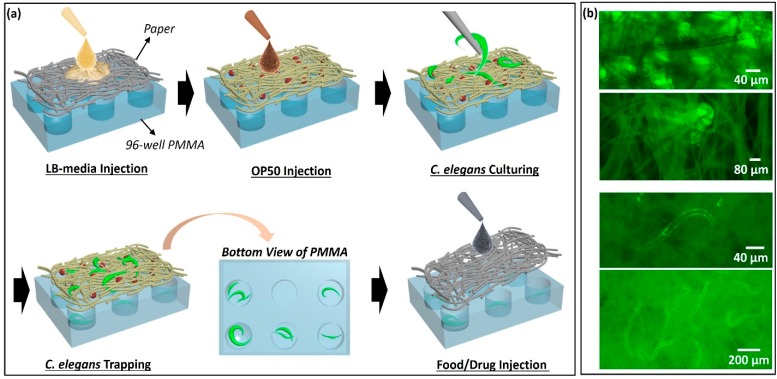
(**a**) Fabrication procedures, (**b**) immobilized *C. elegans* in paper substrates.

In this work, we demonstrated a 3D, high-throughput paper-based platform to realize all culturing, trapping, and monitoring functions for *C. elegans*. Spatially-distinct 96 wells of an array were prepared by sandwiching a filter paper substrate for *C. elegans*’ habitats and micro-machined polymethyl methacrylate (PMMA) supporting frames for high-throughput chambers (Figure 1). After the paper was filled with Luria-Broth (LB) media, the *E. coli* strain OP50 was introduced as a food source for the worms. We then transferred *C. elegans* at the first larval stage (L1) to the paper and cultivated them (Figure 2a). Once their body size reached the pore size of the paper, the *C. elegans* were trapped with low movement capability (Figure 2b,c). 

**Figure 3:**
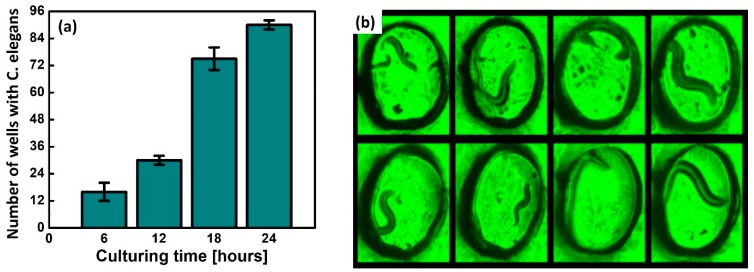
(**a**) Number of wells filled with cultured worms and (**b**) microscopic images of trapped *C. elegans*.

We used *C. elegans* with the reporter genes green fluorescent protein (GFP) for visualization. The transparency of *C. elegans* allowed for microscopic analysis in vivo. After 24 h cultivation, a sufficient number of the worms were reproduced and spread over the paper substrate. Each well contained at least one worm for further experiments (Figure 2a and Figure 3a,b). The porous structure of paper allowed *C. elegans* to move and behave freely in 3D and promoted the efficient growth of *C. elegans* and their primary food, *E. coli* bacteria. When *C. elegans* becomes larger than the pore size of the paper (~10 μm), the worms are trapped and immobilized (Figure 2b). As the worm body size is typically used as a physiological marker to identify size-related phenotype [18], controlling and choosing different pore sizes of paper can be a powerful technique to analyze the potential biophysical and biochemical mechanisms of *C. elegans* at a different larval stage. 

### 2.2. Transparent 3D Culture Systems for C. elegans

**Figure 4:**
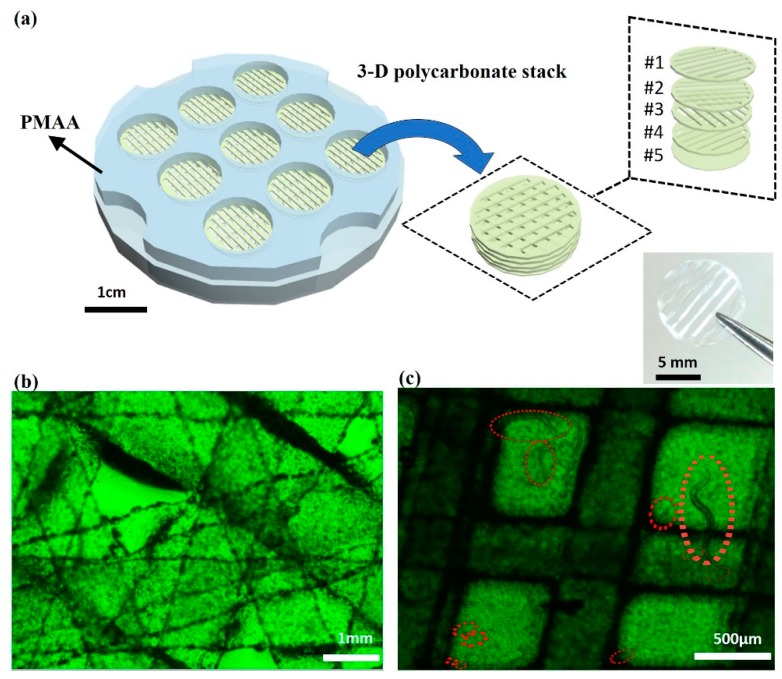
(**a**) Schematic diagrams of the 3-D culture system for C. elegans and microscopic images of the culture regions (**b**) without and (**c**) with *C. elegans*.

Although we visualized the trapped *C. elegans* by using the GFP (Figure 2b), the opacity of paper did not let much light pass through and did not provide high-resolution images in 3D. To enable direct and visual observation, we used paper-like transparent polycarbonate substrates for the culture system [19]. The system used the multi-laminate structure of the substrates to mimic the worm’s in vivo 3D environment. Since the substrate is thin, mechanically strong, and largely porous, the system is the same as our previous one with paper (in Section 2.1) but is transparent to facilitate visualization of the worms under a microscope (Figure S1). The system was constructed by stacking five polycarbonate layers patterned with cuts. Each layer had differently patterned cuts that were perpendicularly crossed over forming small square-shaped through-holes (~500 × 500 μm) at the intersections (Figure 4a). The worms could move vertically through the stack and horizontally along the grids during cultivation. The thick stack provided a 3D aquatic environment holding sufficient amount of LB media for the worms and *E. coli*. The stack was designed to have nine culturing wells (~1 cm diameter) with two PMMS supporting frames (Figure 4). The capillary wicking properties of porous polycarbonate substrates enabled controlled and uniform mass transport of nutrients and gasses for *C. elegans* while their optical transparency allowed real-time monitoring under the microscope without the GFP expression (Figure 4b,c). Moreover, this thin, cost-effective, and disposable polycarbonate substrate permits easy cleaning, sterilizing, and microfluidic pathways. Finally, the stack can be assembled in modular fashion, which allows us to control the thickness of the overall construct and diffusion of nutrients through the stack. Reversely, the simple de-stacking can promote easy *C. elegans* sampling for further experiments.

### 2.3. 3D Behavioral Dynamics of C. elegans

**Figure 5:**
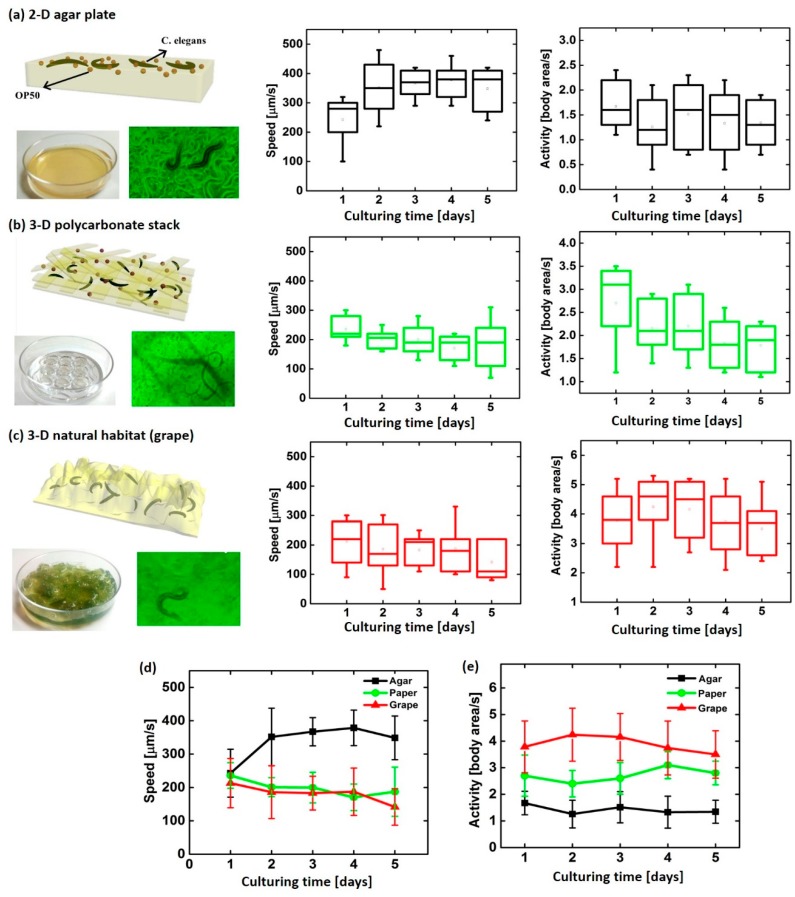
*C. elegans*’ speed and activity (**a**) on 2-D agar plate, (**b**) in 3-D polycarbonate stack, and (**c**) in 3-D natural habitat (grape). Summary of their (**d**) speeds and (**e**) activities.

To demonstrate the performance of the 3D culture system, we prepared three different culturing systems (i) conventional 2D agar plate, (ii) 3D polycarbonate stack, and (iii) 3D natural habitat (i.e., grape) and compared *C. elegans*’ activity and average speed (Figure 5) in each culturing system. The *C. elegans*’ movements were recorded to measure their average speed and the area that the worm crawls about. The grape provided a transparent culturing environment enough to efficiently analyze the worms’ movements. In particular, the worms demonstrated vertical bending activities and movements in 3D platforms while their predominant activity is a horizontal bending on the 2D agar plate. Figure 5 shows the normalized average areas and speeds of *C. elegans* in three environmental systems for five days of culturing, which were measured by WormLab®3.0 software (MBF Bioscience, Williston, VT, USA). The worms on the 2D agar plate showed much higher speed (≥ 350 µm/s) with a smaller moving area because of their limited horizontal bending activity (≤ 2 body area/s) than other 3D systems. The area that the worms moved per second in the grape and in the polycarbonate stack was ~4.2 and ~3.0, respectively, which is much higher than that of the 2D agar plate. Our 3D polycarbonate culture system showed very similar moving activities and speed to the in vivo fruit environment. Interestingly, the worms’ movement speed on the 2D platform was lower at the beginning of culture (Day 1) compared to those on following days after creation of paths throughout the plate while the other 3D systems have similar speed through the culturing days. This indicates that the 2D platform cannot simulate the actual in vivo environment. 

### 2.4. C. elegans’ Growth in 3D Culture System

**Figure 6:**
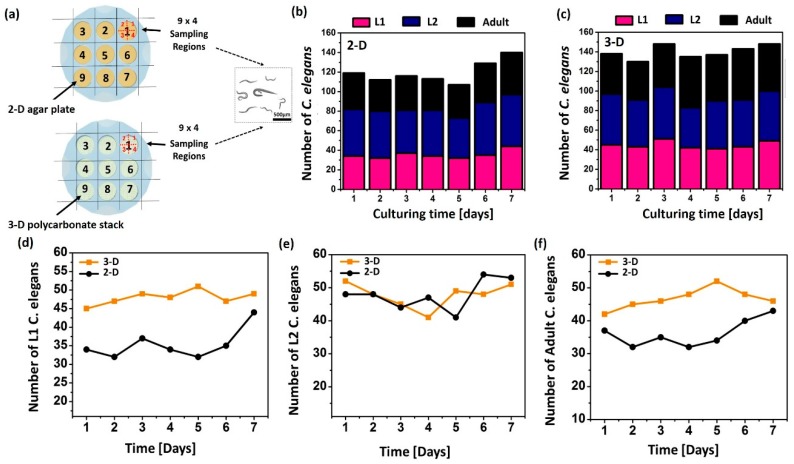
(**a**) Schematic illustration of experimental setup with two culture platforms. The number of *C. elegans* in L1, L2, and adult development stage (**b**) on the 2-D agar plate and (**c**) in the 3-D stack. Summary of the data with (**d**) L1, (**e**) L2, and (**f**) adult worms.

The stereological cell counting method was used to evaluate the worm proliferation in two different 9-well culturing platforms: 2D agar plate and 3D polycarbonate stack (Figure 6a) [20]. Each well was divided into four regions for better microscopic observation. First, we dropped the *E. coli* OP50 onto the culture platforms. Then, *C. elegans* at different growth states were transferred to each well and their development and fertility were observed. For better characterization, *C. elegans* at the L1, L2, and adult stage were counted (Figure 6b–f). Overall, the reproduction and growth rates in the 3D system were higher than those on the 2D plates. This is mainly because the 3D culture platform reproduces more dynamic physiological conditions of the *C. elegans* and provides a more effective environment for nutrient and gas supply. After culturing the worms for seven days on the 3D polycarbonate stack, the individual five layers of the platform were de-stacked to determine the distribution of the worms. Figure S2 confirms the vertical distribution of the worms, indicating that our platform forms a layered structure as a scaffold to offer the in vivo 3D environment. 

## 3. Conclusions

This study reports innovative paper and paper-like platforms for 3D culturing, immobilizing, and monitoring of *C. elegans*. The device was readily fabricated by stacking multiple layers of paper or paper-like polycarbonate substrates. A 96-well paper array was first developed to provide high-throughput culturing environments and in situ monitoring of the worms. Micro-pores of paper allowed *C. elegans* to pass through the paper structure and to be cultivated in 3D. As the worms grow and their body size become comparable to the paper pores, they can be trapped and immobilized by the paper matrix, which eases further high-throughput imaging and analysis. We also created a novel 3D cultivation platform for *C. elegans* by using a paper-like polycarbonate substrate. The transparency of the polycarbonate layers facilitated visualization of the worms under a microscope and allowed us to observe their behavioural dynamics in 3D. Well-defined through-holes in the 3D polycarbonate stack provided a micro-scale in vivo-like and well-controlled environment for *C. elegans*, improving their physical movements, growths, and proliferations. Our technique will offer powerful tools for high-throughput worm-on-a-chip manipulation and fundamental and drug discovery research in *C. elegans*.

## 4. Experimental Sections

### 4.1. C. elegans and Culture

Wildtype Bristol N2, age-1 mutant (TJ1052), and sir-2.1 mutant (VC199) nematode worms were obtained from the Caenorhabditis Genetics Center. All worms were maintained on nematode growth medium (NGM) agar plates containing abundant OP50 Escherichia coli cells as food at 20 °C according to a standard protocol [21]. To produce age-synchronized worms, gravid adult hermaphrodite worms were transferred onto test systems for culturing and comparison studies.

### 4.2. Preparing E. coli OP50 Bacteria

Escherichia coli OP50 were grown from −80 °C glycerol stock cultures by inoculating 20 mL of L-broth medium with gentle shaking in air for 24 h at 35 °C. The L-broth media consisted of 10.0 g tryptone, 5.0 g yeast extract, and 5.0 g NaCl per liter. The culture was then centrifuged at 5000 rpm for 5 min to remove the supernatant. The bacterial cells were re-suspended in a new medium and used as a feeding inoculum for culturing systems.

### 4.3. A Paper-Based 96-Well Culturing Platform

The platform consisted of four functional layers as shown in Figure 1; a Whatman #1 filter paper, a PMMA boundary layer, a PMMA 96-well layer, and a PMMA bottom layer. Each layer was micro-patterned by using laser micromachining (Universal Laser System, VLS3.5, Yokohama, Japan). All transparent PMMA layers were thermally bonded at 100 °C for 1 h. The worms reproduced and were collected in each well while the fully-grown ones were trapped and immobilized by small paper pores. 

### 4.4. A Transparent 3D Culture System

We chose paper-like polycarbonate as a scaffold for the model environment for *C. elegans* culturing. Since the polycarbonate layer is thin, transparent, mechanically strong, and soft, it is suitable for forming a 3D structure for cell culture. Polycarbonate layers have been widely used as an excellent bio-scaffold for cell distribution, adhesion, and growth, as well as allowing for fluorescence imaging without light scattering [19,22,23]. The PMMA layers and polycarbonate substrates were precisely cut by a laser cutting machine. All layers were aligned and assembled to define 96 wells for culturing (Figure 4). 

### 4.5. Stereological Cell Counting

Counting the number of cells in a region of interest with stereology yields reliable quantitative data that can be used to determine if the number of cells differs between experimental and control groups [20]. In this work, we used a systematic random sampling (SRS) method to obtain a statistically valid sample of the region of interest (Figure 4). We divided each culturing well into four regions and recorded the quantified data. 

### 4.6. Fertility and Distribution Analysis

The growth and development of multicellular organisms are critically linked to their nutritional status [24]. The four distinct larval stages (L1–L4) during *C. elegans* growth provide easily detectable developmental milestones. In this study, we considered the number of *C. elegans* at L1, L2, and adult stage as a critical indicator of culturing yield (Figure 6). After seven days of culturing, the layers were disassembled and the number of worms in 36 randomly selected regions were counted (Figure S2).

## Supplementary Materials

The following are available online at https://www.mdpi.com/2072-666X/11/1/99/s1. Figure S1: (a) SEM and (b) microscopic image of polycarbonate; Figure S2: Number of C. elegans distributed in the individual layers.
