# Supplementary material for: Paper-Supported High-Throughput 3D Culturing, Trapping, and Monitoring of Caenorhabditis Elegans
Source: Micromachines (Basel). 2020 Jan 17;11(1):99. doi: 10.3390/mi11010099 (PMC7020171; doi:10.3390/mi11010099)
Supplement: Supplementary file 1 [file micromachines-11-00099-s001.pdf]

micromachines

Supplementary Materials: Paper-Supported High-Throughput 3-D Culturing, Trapping, and Monitoring of Caenorhabditis Elegans

Mehdi Tahernia, Maedeh Mohammadifar and Seokheun Choi

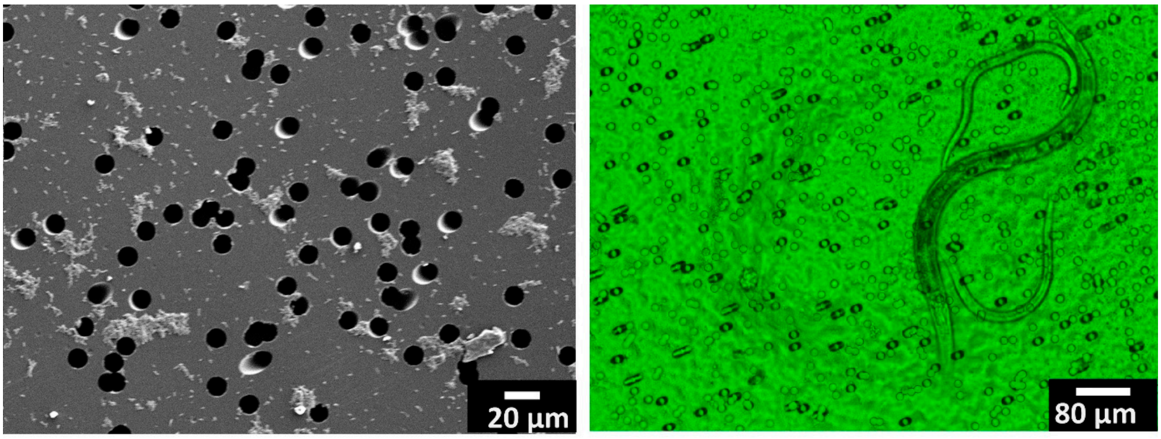

Figure S1. (**a**) SEM and (**b**) microscopic image of polycarbonate.

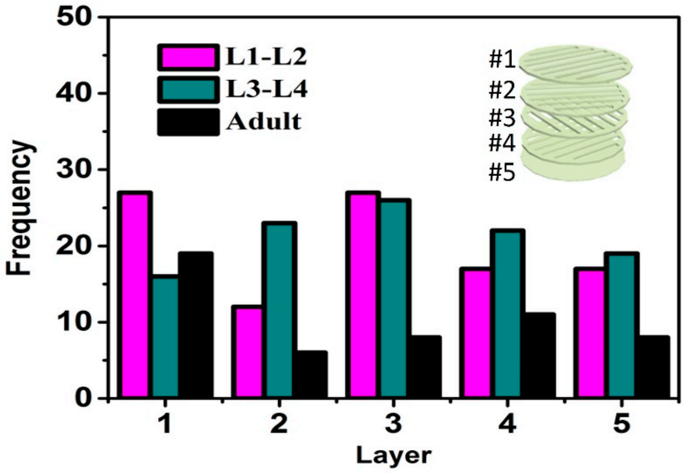

Figure S2. Number of C. elegans distributed in the individual layers. .
